# Incidence and etiology of traumatic spinal cord injury in Rwanda: a prospective population-based study

**DOI:** 10.3389/fneur.2024.1373893

**Published:** 2024-08-21

**Authors:** Maurice Kanyoni, Lena Nilsson Wikmar, Joliana Philips, Conran Joseph, David K. Tumusiime

**Affiliations:** ^1^University of Rwanda College of Medicine and Health Sciences, Kigali, Rwanda; ^2^Department of Neurobiology, Care Sciences and Society (NVS), Division of Physiotherapy, Karolinska Institutet, Stockholm, Sweden; ^3^Department of Physiotherapy, University of the Western Cape, Bellville, South Africa; ^4^Faculty of Medicine and Health Sciences, Department of Health and Rehabilitation Sciences, Division of Physiotherapy, Stellenbosch University, Cape Town, South Africa

**Keywords:** spinal cord injury, incidence, Rwanda, LMIC, Africa

## Abstract

**Background:**

Traumatic spinal cord injury (TSCI) is not only a life-threatening but also life changing event that happens suddenly, the effects extends beyond the TSCI survivors to include their families. In Rwanda to the best knowledge of authors, there is no published information on the epidemiology of TSCI. The aim of this study was therefore to determine the incidence rate, etiology and injury characteristics of TSCI.

**Methods:**

All survivors of acute TSCI who met the inclusion criteria were prospectively recruited for a one-year period. The International Spinal Cord Injury Core Data Set was used to collect the minimum set of variables to facilitate worldwide comparison of epidemiological data, while the International Standards for the Neurological Classification was used to categorize TSCI according to the American Spinal Injury Association Impairment Scale (AIS). Data were collected by trained physiotherapists.

**Study design:**

A prospective, open-ended, cohort study design.

**Setting:**

All referral hospitals within the Republic of Rwanda.

**Results:**

Overall, 122 adult individuals sustained a TSCI between 10th October 2019 until 9th October 2020 and all consented to take part in the study. The male-to-female ratio was 3.9:1, and the mean age was 42.5 (SD = ±14.8) years. The crude incidence rate of TSCI was 22.2 per million people (95% CI, 18.4–26.5) with significant differences in sex-adjusted rates for all age groups while men 46 years of age and older presented with the highest incidence. The leading causes of TSCI were falls (73.8%), followed by road traffic accidents (18.9%). Moreover, SCI lesions of the cervical region (*n* = 69) were the most common, followed by the lumbosacral region (*n* = 27). Fifty-one (41.8%) participants were diagnosed as complete injury, i.e., AIS A, while incomplete injury category C constituted 35 (28.7%).

**Conclusion:**

The incidence and etiology of TSCI in Rwanda are comparable to worldwide estimates and figures. Largely, the etiology of TSCI are preventable as it is caused due to falls and road traffic accidents. There is a need to consider preventive strategies and policies on activities that predispose people to falls. Policies should focus largely on occupational health and safety in both formal and informal sectors of work.

## Introduction

Traumatic spinal cord injury (TSCI) is a life-threatening event that happens suddenly and not only affects victims of injury but also their families (1). The challenges that survivors of TSCI face depend on several factors, for example, the level and completeness of injury, and facilitators and barriers in their respective environment. A systematic review of studies carried out in 2018 by Kumar et al. (2) found that the incidence of TSCI was higher in low and middle-income countries (LMICs), at 13.7 per 100,000 persons, compared with high income countries of 8.7 per 100,000 persons (2). The same review found the global incidence of TSCI to be 10.5 cases per 100,000, while road traffic accidents, followed by falls, were the leading causes of TSCI worldwide, and that globally males were more affected than females at a ratio of 3:37, with the highest gender ratio 7:35 (M/F) found in Brazil, South America.

A systematic review of 2017 set out to establish the incidence of TSCI in Middle East and North African countries (MENA) and reported an estimated regional incidence between 7.8 per million in Kuwait and 72.45 per million in Iran (3). The same review reported a pooled regional incidence of 23.4 per million. The sub-Saharan regional incidence of TSCI is not well documented as alluded to by Draulans et al. (4), and where studies are available, they used retrospective designs, and their catchment area is limited to a particular region or individual hospital. However, in recent years, there are a number of individual country studies on the epidemiology of TSCI in sub-Saharan Africa, with incidence rates for South Africa reported at 75.6 per million (5), North East Tanzania at 38 per million (6), and Botswana at 13 per million people (7). One consideration for the divergence of the aforementioned incidence rates could be attributed to only adults (18 years and above) being included in the South African and North-East Tanzanian studies while the entire population (children and adults) was included in the Botswanan study. A single hospital-based study carried out in Malawi reported 46 newly-injured patients in a 9-month surveillance period (8).

There are limited emergency services in developing countries, and it is rare that a person with TSCI is immobilized and transported by trained personnel. This can worsen neurological symptoms in cases of an unstable spine. Delays in transport to convey patients from the injury site to a health care facility with specialized services required by a person with TSCI in low- and middle-income countries (LMIC) is another risk factor for poorer vital and neurological outcomes (9).

Rwanda lacks published information on the epidemiology of TSCI, which hinders the development of primary prevention campaigns as well as strengthening of rehabilitation services to facilitate recovery and independence in normal life routines. The aim of this study was therefore to determine the incidence rate, etiology and injury characteristics of TSCI.

## Materials and methods

### Design

A prospective, open-ended, cohort study design was used. All acutely injured survivors of a TSCI fulfilling the inclusion criteria were prospectively included in a one-year cohort study from 10th October 2019 to 9th October 2020.

### Study setting

The study was carried out in Rwanda, one of the smallest countries on the African continent. According to the last census of 2012, the Rwandan population is estimated at 10,515,973 people spread across an area of 26,340 km^2^ (10). The total number of people aged 18 years old and above, as per the same census, is estimated at 5,500,845 people (10).

The healthcare system in Rwanda is designed in what can be regarded as a down-up pyramid with a health center as the primary health facility and a national referral hospital as the most tertiary facility (11). There are intermediate facilities; district hospitals and regional hospitals. There are four national referral hospitals in the country which offer specialized services that a patient with TSCI may require. These referral hospitals were all included in the study. Three of these hospitals, i.e., Kigali University Teaching hospital (KUTH), Rwanda Military hospital (RMH) and King Faisal hospital Kigali are situated in central Rwanda, while Butare University Teaching hospital (BUTH) is located in the southern part of the country. The four hospitals receive patients either directly or on a referral basis from the whole country. As a guideline, trauma patients are received at the emergency unit, where appropriate assessments and examinations are done. When a surgeon confirmed a TSCI, a patient is transferred to the trauma unit of the same hospital, or one of the four referral hospitals.

### Inclusion criteria

Inclusion criteria for this study were: (1) admission to one of the four hospitals; (2) survival for at least 7 days after the initial injury; (3) confirmed (by magnetic resonance imaging or computer tomography/CT scan) primary diagnosis as acute traumatic spinal cord or cauda equina lesion; (4) aged 18 years and older at the time of injury; (5) a resident of Rwanda at the time of data collection; and (6) patients who provided consent. Excluded were people who sustained TSCI before 10th October 2019 because it was a one-year incidence, those below the age of 18 years old because under the Rwanda law they are regarded as minors.

### Data collection, procedure and ethics

The study protocol was approved by the Institutional Review Board of the College of Medicine and Health Sciences at the University of Rwanda (Approval No. 308/CMHS IRB/2019). Those who agreed to take part voluntarily signed a consent form. We certify that we complied with the principles in the Helsinki declaration in its amendments up to date and the Ministry of Health, Republic of Rwanda guidelines on human research participants, before, during and after data collection.

The instrument for data collection consisted of the International SCI Core Data Set, as recommended by the members of the Executive Committee for the International SCI Data Sets Committees (12). Variables included: date of birth, gender, date of injury, date of acute admission and discharge, etiology, presence of vertebral fractures and associated injuries; associated injuries for the purposes of this study were defined as those that occurred at the same time as the spinal cord injury. These are moderate to severe traumatic brain injury (Glasgow Coma Scale 12 or below at discharge), non-vertebral fractures requiring surgery, severe facial injuries affecting sense organs, major chest injury requiring, traumatic amputations of an arm or leg, severe hemorrhage, or damage to any internal organ requiring surgery. We further documented whether spinal surgery was performed, ventilator-dependence of patients at discharge and place of discharge. Assessment of neurological severity was conducted using International Standards for Neurological Classification of Spinal Cord Injury (ISNCSCI) (13). The use of ISNCSCI and International SCI core data set in clinical practice and research in Rwanda is new. We therefore conducted a two-day training of two Physiotherapists at each research setting/hospital on the use of the tools. We conducted a one-day pilot at one of the hospitals; Rwanda military hospital to assess whether the trainees are conversant with the tools. Thus, each team of two physiotherapists at each of the four hospitals completed the International SCI Core Data Set for all the consenting patients. Data collection started on 10th October 2019 and ended 9th October 20220.

### Data analysis

Study participants’ profile details such as age and gender, injury characteristics, severity and etiology were analyzed descriptively using IBM Statistical Package for Social Sciences (SPSS version 26, IBM, SPSS, New York, United States). Continuous variables were presented as mean, standard deviation (SD) and median. Categorical variables were expressed as number of cases and percentages. For the age variable, 15 year increments were used as recommended by the International Spinal Cord Injury Scientific committee on education and research (14). Differences between sub-groups were analyzed using Chi Square Test. The incidence rate was calculated based on the current country population figures at the time, excluding those that are under the age of 18 years old. The indirect standardization method was used whereby the nominator reflected the number of TSCI cases in 1 year (*n* = 122) and the denominator reflected the person-time, which was 5,500,845 (all aged 18 and above). Furthermore, incidence rate differences were calculated for sex- and age-adjusted sub-groups, and Fisher’s exact estimations with 95% confidence intervals were used since some sub-groups contained fewer than five cases.

## Results

### Participant characteristics

Overall, 122 individuals sustained a TSCI during the study period and consented to be included in the study. The male to female ratio was 3.9:1 with 80.3% (*n* = 98) males and 19.7% (*n* = 24) females. The mean age at the time of injury was 42.5 (±14.8), ranged from 18 to 72 years, with most injuries in the 46–60 age category *n* = 39 (32%). In total, 83.6% (*n* = 102) sustained vertebral injuries and 34.4% (*n* = 42) underwent spinal surgery. Associated injuries were present in 33.4% (*n* = 41) of the cohort. Table 1 summarizes the participant characteristics.

**Table 1:** Participant characteristics (*n* = 122).

Variables			Frequency (n)		Percent (%)
**Gender**					
Male			98		80.3
Female			24		19.7
**Age at injury in years**					
18–30			34		27.9
31–45			34		27.9
46–60			39		32
≥ 61			15		12.3
Average age (SD)			42.5 (14.8)		
**Spinal surgery**					
Yes			42		34.4
No			80		65.6
**Vertebral injury**					
Yes			102		83.6
No			20		16.4
AIS*					
A			51		41.8
B			5		4.1
C			35		28.7
D			31		25.4
AIS*		A (n)	B (n)	C (n)	D (n)
**Lesion level**	C1-C4	18	0	8	8
	C5-C8	14	0	14	7
	T1-T12	15	3	5	3
	L1-S5	4	2	8	13

*American Spinal Injury Association (AIS) Impairment scale; A, Complete; B, Sensory Incomplete; C, Motor Incomplete; D, Motor Incomplete (9).

### Crude and age- and gender-adjusted incidence rate

At the time of data collection, Rwanda’s total population was 10,515,973 people, of which 5,500,845 people were 18 years old and above. The 122 newly-injured cases in 1 year of our study translates to a crude incidence rate of 22.2 per million person-time (95% CI: 18.4–26.5). A significant difference was found in the incidence rates between men and women, estimated at 38.0 per million (95% CI: 31.0–46.3) and 8.2 per million (95% CI, 5.3–12.2) respectively. Furthermore, significant differences in incidence rates were found in all age groups, i.e., 18–30; 31–45; 46–60; 61 and more, in comparison to gender with men demonstrating a higher incidence rate across all age categories. The largest differences in incidence rates between men and women were for the older age groups, specifically categories 46–60 and 61 and more as reflected in Table 2.

**Table 2:** Traumatic spinal cord injury incident cases, age and gender-specific incidence rates (per 1,000,000 annually) with 95% confidence intervals (CIs) for males, females and total population in Rwanda.

Age groups	No.	Rates (males)	95% CI	No.	Rates (female)	95% CI	*p* value	No.	Rates (All)	95% CI
18–30	29	22.8	15.3–32.8	5	3.77	1.2–8.8	**<0.0001**	34	13.1	9.1–18.3
31–45	22	30.6	19.2–46.3	12	14.5	7.5–25.4	**0.02**	34	22.0	15.2–30.8
46–60	36	88.8	62.2–122.9	3	6.0	1.2–17.6	**<0.0001**	39	43.1	30.6–58.9
≥61	11	60.0	30.0–107.2	4	14.6	4.0–37.5	**0.005**	15	32.8	18.4–54.2
**Total**	98	38.0	31.0–46.3	24	8.2	5.3–12.2	**<0.0001**	122	22.2	18.4–26.5

Bold figures stands for significant values (*p* < 0.05).

### Etiology

**Figure 1:**
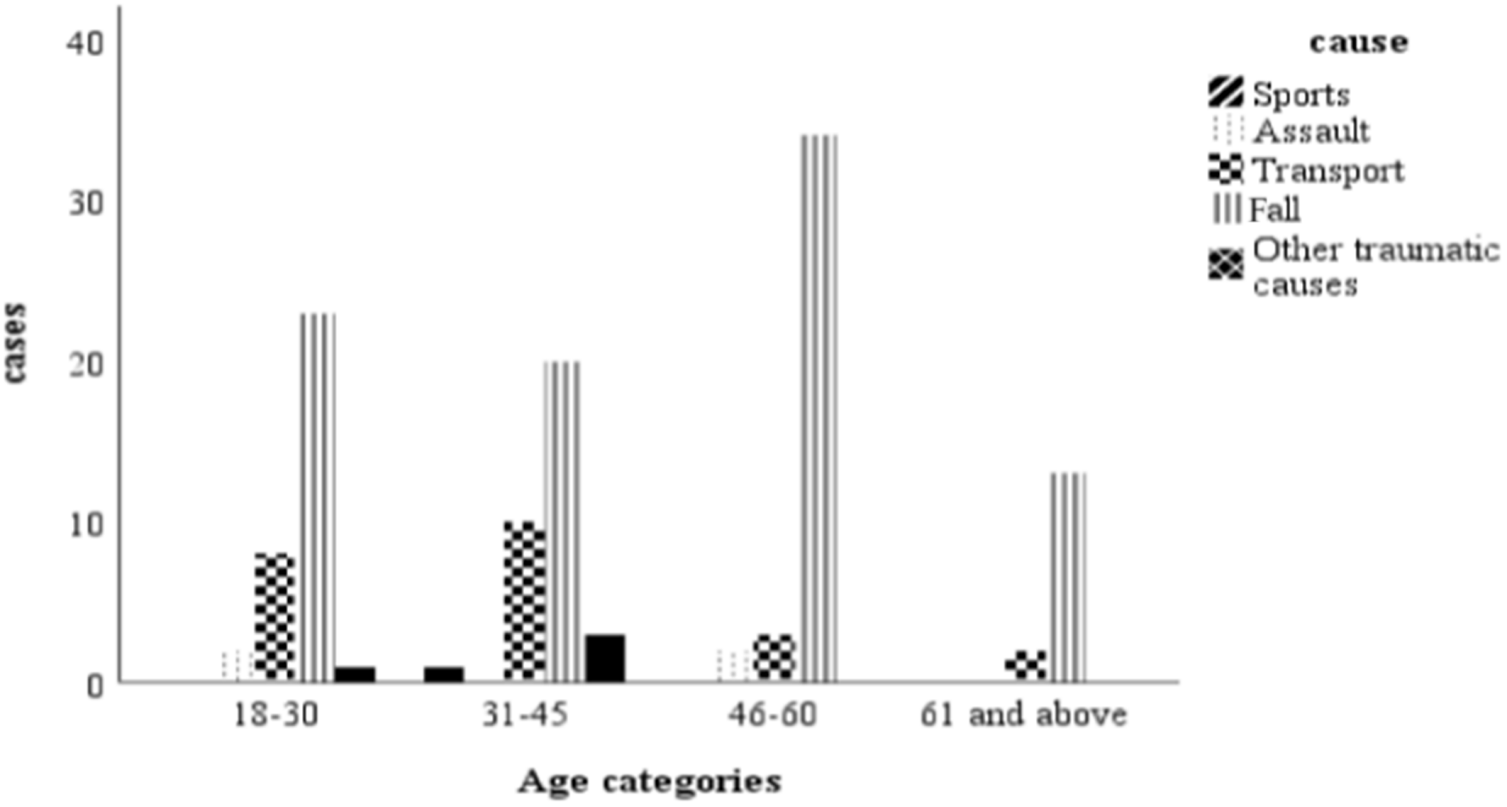
Number of participants and different causes of traumatic spinal cord injury along age spectrum.

The most common cause of TSCI in this study was falls, which accounted for 73.8% of injuries, followed by road traffic accidents at 18.9%. Fall-related injuries were more common in males (*n* = 75; *p* = 0.039) than females (*n* = 47). Falls were the most frequent cause of injury in all age categories, affecting mostly those between 46 and 60 years, followed by those between 18 and 30 years. Road traffic accidents were the second most frequent cause of TSCI and affected more men than women (*n* = 17; *p* = 0.02). Sport-related TSCI were relatively few compared to other causes, as shown in Figure 1.

### Location and severity of injury

**Figure 2:**
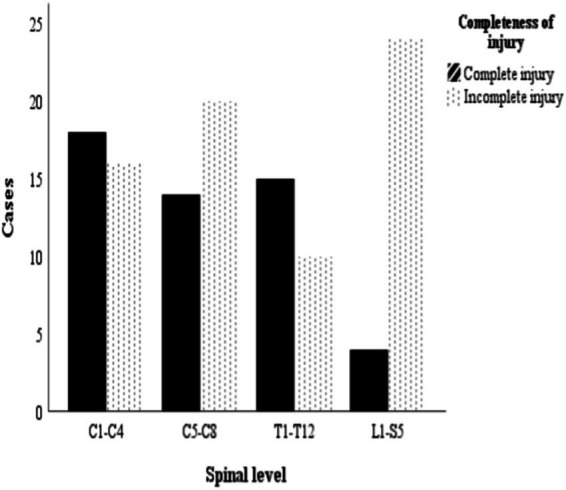
Number of participants in relation to location of traumatic spinal cord injury and severity.

Figure 2 illustrates the location and severity of injury among study participants. The study registered more incomplete spinal cord injuries than complete injuries in proportions of 58.2% (*n* = 71) and 41.8% (*n* = 51) respectively and this difference was significant (95%, CI, 1.82–2.38; *p* = 0.003). Overall, most of the participants presented with injuries in the cervical region (C1-C8, *n* = 69; 56.6%) on admission, followed by the lumbosacral region (*n* = 27; 22.1%), while those whose injuries were in the thoracic region constituted *n* = 26; 21.3%, (Table 1). Fifty-one (41.8%) participants were diagnosed as complete injuries, with 32 (62.7%) occurring in the cervical region, 15 (29.4%) in the thoracic region and 4 (7.8%) in the lumbosacral region. Participants classified as AIS C constituted the second largest proportion (*n* = 35) with the highest proportion identified in the cervical region (*n* = 21). Seventy-one participants (58.2%) sustained incomplete spinal cord injury.

## Discussion

To our knowledge, this is the first epidemiological study done in Rwanda on the epidemiology of TSCI. The main findings suggest that the incidence rate, at 22.2 per million, is comparable with other African and global settings. Secondly, the leading cause of injury was due to high falls in predominantly males, and a large proportion of injuries result in complete (AIS A) lesions. This information should be used to target primary preventive interventions in Rwanda.

The country incidence of 22.2 per million people is comparable to what was found in a systematic review of studies conducted for the Middle East and North Africa (MENA) region, with an estimated regional incidence of 23.4 per million people (3). Similarly, the incidence of TSCI in the current study lies within the Africa region which ranges from 21 (Sub Saharan Africa Central) to 29 (Sub Saharan Africa East) per million people (15). However, the incidence of TSCI in Rwanda is lower than that of a previous South African report which found an incidence of 75.6 per million people (5), and North East Tanzania with an incidence of 38 per million people (6). One possible explanation of the difference in incidence rate reported in this study compared to other African countries is road transport safety policies that are uniquely found in Rwanda compared to the rest of Africa like strict use of speed governors in all public transport buses, speed cameras, compulsory use of seat belts and relatively low speed limits on the roads; most of the time 6 km/h. This shows how policies can vary risk factors and save life in Africa.

With respect to more global comparison, the Rwandan incidence rate is by far lower than the global incidence of 105 per million people and that of low-income countries of 137 per million people (2). TSCI poses a direct, significant burden to society due to its associated impact on the health system, individuals and families, as well as indirect cost due to lost productivity imposed by morbidity and premature death. In recent years, Rwanda has invested significantly in healthcare, among others: training of personnel, strengthening emergency care services and health financing (16), but insufficient specialized services for TSCI patients still exists. Considering the consequences of TSCI and the human and financial resources to deal with the artifacts of it, there is a need to prevent TSCI in Rwanda.

Other than a limited number of countries in the north and south of Africa, limited data from the rest of Africa are available which directly hinders efforts to curb the occurrence of TSCI on the continent. The most frequently reported cause of TSCI in Rwanda was found to be falls and this is consistent with what was highlighted in a recent systematic review on the leading causes of TSCI in low-income countries (2). Similarly, falls was also mentioned as the leading cause of TSCI in the North-East Tanzanian study (6). Mechanism of injury was not among the aims of this study but rather epidemiological data because there is a gap for this in Rwanda. It could therefore be speculated that falls are due to either falling while carrying heavy loads on the head, agricultural activities, especially rice growing, mining and landslides. However, Rwanda’s pattern is likely to change in future due to transitioning to motorized road transportation which may bring about improved employment and trading opportunities for its citizens. The leading causal factors of TSCI in Rwanda differs from causes elsewhere in Africa; countries like South Africa reported assault, (5) while Botswana (7) and Malawi (8) report road traffic accidents as the leading cause of TSC. The differences in causative factors might be explained by differences in socio-economic, political (policies) and geographical reasons. For example, in Rwanda there are strict laws against acquiring and handling of elicit weapons other than security personnel. Strict enforcement of road safety policies like heavy penalties for drunk driving, installation of speed cameras and speed governors also might be an explanation of observed low road traffic accident causes.

Our study showed that males were most affected than females which is similar to other developed contexts as well as low- and middle-income countries (2). These gender disparities in terms of rates of TSCI was reported in other studies (5–7). This trend is observed in some middle income countries, however, in the developed world, TSCI affect more individuals 65 years and older (15). This gender profile of TSCI in Rwanda is also reflected in other African studies; a South African study found that 85.5% were male, (5) in a study in Botswana 71% male (7), and in a study in North-East Tanzania found a proportion of 79.3% male (6). This observable gender profile in Africa could be attributable, in part, to males being more involved in productive activity, them driving more often than women, and males engaging in more risk-taking behaviors. Furthermore, spinal cord lesions occurred more in the cervical region which is also observed globally (2). This SCI characteristic was also reported in South Africa (53.1%) (5), Botswana (59%) (7), and North-East Tanzania (56.3%) (6). This clearly shows the disability burden faced by TSCI survivors since the higher level lesions often result in more pronounced functional and health related challenges.

### Strengths and limitations

The prospective collection of data in this study was a strength in that all exposures (causes, baseline data variables) and outcomes (TSCI) were recorded from the outset. The study was conducted at four referral hospitals where SCI patients are referred to for treatment, indicating that the entire population was under surveillance and part of the study population. Data collection was carried out by the same team throughout the period of data collection, hence reliability of data is enhanced. Internationally validated outcome measures were used for patient assessment which afford direct comparison.

Limitations of the study is that a number of TSCI patients (who may be very mildly affected) failed to reach national referral hospitals due to a variety of reasons. There might be TSCI patients who might have passed away during the course of the study, but specifically before they were assessed and included in the study. The impact of the COVID-19 pandemic also limited the study; the first case of COVID-19 was reported mid-way through the study period and a national lockdown was announced in the sixth month of data collection. Therefore, the study results need to be interpreted in light of the wider influence of the pandemic on regulations on traveling and mobility. The other combined impact of COVID-19 and pre-admission mortality would be underestimation of TSCI incidence in Rwanda. The registration was for a period of only 1 year, and therefore annual fluctuations could not be taken into account.

## Conclusion

This study shed necessary light on aspects of the epidemiology of TSCI in Rwanda. The incidence rate of 22.2 per million persons is in line with other countries in Africa and the world-at-large. Interestingly, the leading cause of TSCI was found to be falls, followed by motor vehicle accidents, which follows a trend that is seen in more developed contexts. However, falls here may be related to occupational duties or opportunities whereby men work in mines, agriculture and climb trees in order to secure food and fire wood. Given the higher proportion of persons with cervical level lesions, which are often complete injuries, a need remains to develop contextually-relevant primary prevention strategies to curb the occurrence of TSCI in Rwanda.

## Data Availability

The raw data supporting the conclusions of this article will be made available by the authors, without undue reservation.
